# Genomic and metagenomic insights into the microbial community of a thermal spring

**DOI:** 10.1186/s40168-019-0625-6

**Published:** 2019-01-23

**Authors:** Renato Pedron, Alfonso Esposito, Irene Bianconi, Edoardo Pasolli, Adrian Tett, Francesco Asnicar, Mario Cristofolini, Nicola Segata, Olivier Jousson

**Affiliations:** 1 Centre for Integrative Biology, University of Trento, 38123 Trento, Italy; 2 Istituto G.B. Mattei, Stenico, Italy

**Keywords:** Shotgun metagenomics, High-throughput culturing, Thermal spring, Comparative genomics

## Abstract

**Background:**

Water springs provide important ecosystem services including drinking water supply, recreation, and balneotherapy, but their microbial communities remain largely unknown. In this study, we characterized the spring water microbiome of Comano Terme (Italy) at four sampling points of the thermal spa, including natural (spring and well) and human-built (storage tank, bathtubs) environments. We integrated large-scale culturing and metagenomic approaches, with the aim of comprehensively determining the spring water taxonomic composition and functional potential.

**Results:**

The groundwater feeding the spring hosted the most atypical microbiome, including many taxa known to be recalcitrant to cultivation. The core microbiome included the orders *Sphingomonadales*, *Rhizobiales*, and *Caulobacterales*, and the families *Bradyrhizobiaceae* and *Moraxellaceae*. A comparative genomic analysis of 72 isolates and 30 metagenome-assembled genomes (MAGs) revealed that most isolates and MAGs belonged to new species or higher taxonomic ranks widely distributed in the microbial tree of life. Average nucleotide identity (ANI) values calculated for each isolated or assembled genome showed that 10 genomes belonged to known bacterial species (> 95% ANI), 36 genomes (including 1 MAG) had ANI values ranging 85–92.5% and could be assigned as undescribed species belonging to known genera, while the remaining 55 genomes had lower ANI values (< 85%). A number of functional features were significantly over- or underrepresented in genomes derived from the four sampling sites. Functional specialization was found between sites, with for example methanogenesis being unique to groundwater whereas methanotrophy was found in all samples.

**Conclusions:**

Current knowledge on aquatic microbiomes is essentially based on surface or human-associated environments. We started uncovering the spring water microbiome, highlighting an unexpected diversity that should be further investigated. This study confirms that groundwater environments host highly adapted, stable microbial communities composed of many unknown taxa, even among the culturable fraction.

**Electronic supplementary material:**

The online version of this article (10.1186/s40168-019-0625-6) contains supplementary material, which is available to authorized users.

## Background

Water springs play an essential role as transition areas (ecotones) between groundwater, surface water, and terrestrial ecosystems [1]. The patchy distribution of such environments results in very specialized biomes, implying that close-by water springs in the same catchment basin may host very different communities [2]. Microbial communities in groundwater and water springs consist of both endemic species and taxa deriving from the surrounding environments [3]. Water leaching through soil and rocks seeds the groundwater with bacteria with very heterogeneous metabolic requirements, but only a few of them are able to survive and thrive in this challenging environment [4]. Groundwater challenges include physical constrains such as the absence of light, making photoautotrophy impossible [5], low availability of organic substrates [5], presence of toxic chemical substances such as xenobiotics or heavy metals, usually derived from rock leaching or carried by water [5], and persistent presence of phages [6]. Most bacterial cells in pristine aquifers live attached to the mineral particles, while planktonic lifestyle is less frequent. Microbial communities and chemical composition of water are known to have an impact on each other. On the one hand, microbial community composition and biomass play a key role in water primary production and electric conductivity [7, 8], especially in alpine settings, where organic matter content is scarce [9]. On the other hand, water chemistry seems to influence microbial community composition and abundance [8, 10].

Traditional culturing, 16S amplicon sequencing and shotgun metagenome sequencing represent well-established methods to study environmental or host-associated microbiomes [11]. When applied in combination, these approaches provide a solid basis for the characterization of both the culturable and unculturable part of the microbiota. A recently introduced method in the field of metagenomics allows the assembly of draft microbial genomes starting from the metagenomic data set. This approach relies on both composition and differential coverage of genomes among samples, implying that metagenome-assembled genomes (MAGs) must be abundant enough so that their genomes can be adequately covered. Recently, this approach has been used in both environmental and host-associated microbiome studies [12, 13]. In the study of Brown et al., 797 genomes belonging to the new super-phylum of *Patescibacteria* (also known as “Candidate Phyla Radiation” or CPR), were assembled from groundwater metagenomes [12]; in the study of Jeraldo et al., the genome of an uncultured species was assembled from the gut microbiome, providing valuable insights about its potential microniche in the host [13].

Conversely, cultivation allows to isolate bacteria with specific metabolic requirements, without the need to be abundant in the original sample; in principle, a single cell or colony-forming unit is sufficient to yield high amount of biomass for subsequent phenotypic and genotypic characterization. Natural microbial communities are usually dominated by a minor fraction of abundant species and a long tail of rare ones [14]. To date, most available culture media and protocols are just enough for the isolation of species belonging to a small number of phyla (e.g., *Proteobacteria*, *Firmicutes*, *Actinobacteria*), which may not be the most abundant ones in some environments [15]. Therefore, isolation efforts are more likely to target species from the low-abundance tail [14].

Water spring ecosystems provide services that go beyond water supply. Human populations worldwide have exploited these environments for recreational, medical, and religious purposes from ancient until modern times. The therapeutic use of spring water is documented in Ancient Egypt, Greek and Roman civilizations, Japan, and China. These practices subsequently spread across Europe and America during the nineteenth century. Nowadays, balneotherapy centers offer treatments for several pathologies, mainly dermatological conditions including psoriasis, acne, atopic dermatitis, contact dermatitis, seborrheic dermatitis, and upper respiratory tract diseases, such as recurrent upper respiratory tract infections, allergic and non-allergic rhinitis, and acute and chronic rhinosinusitis [16–18].

In this study, we integrated culturomics and metagenomics approaches to provide, to our knowledge, the first ever comprehensive view of a spring water microbiome. We describe the taxonomic composition of bacterial communities of the thermal spring of Comano Terme (Italy), as well as the genomes obtained either from cultivation of isolates or from the metagenomic dataset, and infer their main functional traits in relation to the spring water environment.

## Results

### Comano Terme spring microbiome composition remains constant throughout the year

The total microbial load of the thermal spring of Comano Terme resulted to be 5·10^6^ cells/l. By cultivation, we built a collection of 181 bacterial isolates, belonging to 4 phyla, 6 classes, and 38 genera (Table 1). Their preliminary taxonomic identification by 16S sequencing revealed that 18 isolates could be classified at the genus level, while 6 isolates were classified at the family level, and 1 isolate was classified at the order level. After filtering out, the OTUs present in the negative-control samples (Additional file 1: Table S1), the 16S amplicon sequencing approach resulted in an average reads number of 498.143 s.d. 224.382, with a range spanning 133.090–724.934 (Additional file 2: Table S2).

**Table 1:** Diversity of the strain collection according to the number of taxa isolated at different taxonomic ranks

Sampling site	Number of isolates	Phyla	Classes	Orders	Families	Genera
Spring	61	3	5	8	10	18
Hydra	59	3	5	8	14	17
Storage tank	31	3	5	6	12	14
Bathtub	30	3	5	7	8	12
Total	181	4	6	12	22	38

**Fig. 1:**
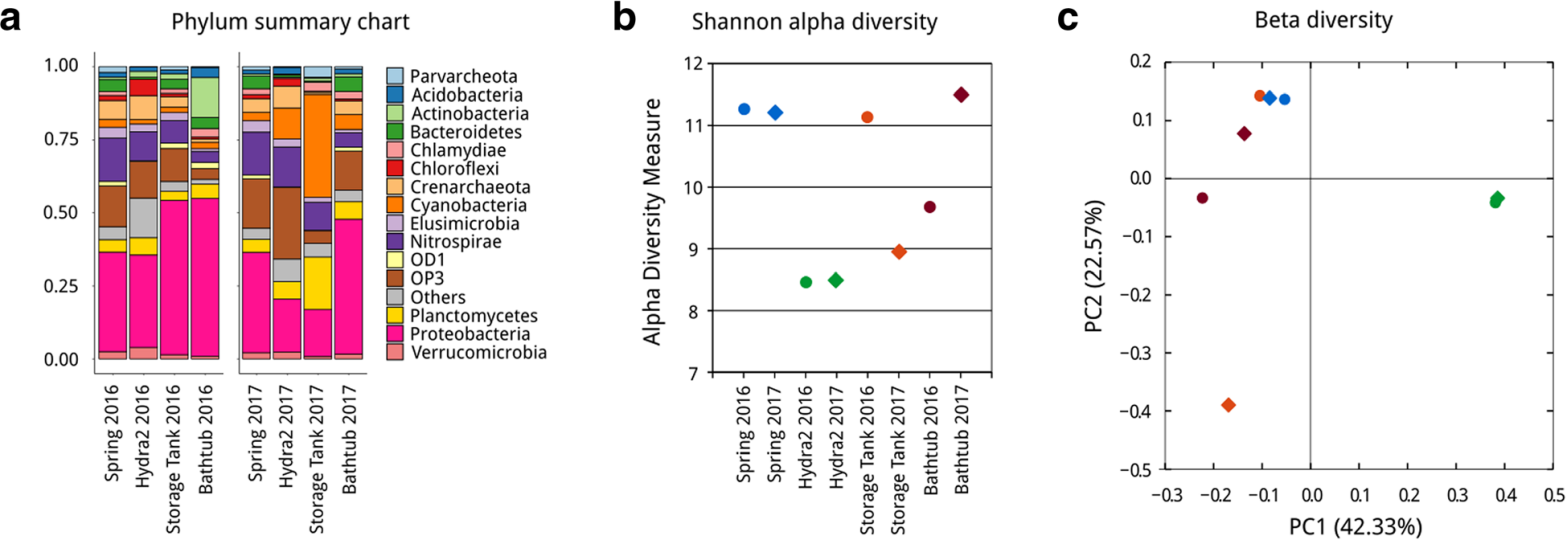
Microbiome composition of Comano Terme water inferred by 16S amplicon sequencing. **a** Relative abundancies at the phylum level. **b** Shannon alpha diversity index values; each sampling point is color-coded as follows: spring, blue; hydra, green; storage tank, orange; bathtub, bordeaux. Samples are shape-coded as dots for 2016 and diamonds for 2017. **c** Beta diversity analysis presented as weighted UniFrac principal-coordinate analysis (PCoA); color and shape coding are the same as for panel **b**

The composition of the microbiome (Fig. 1a) is dominated by *Proteobacteria* (35.9% s.d. 14.4%) and *Nitrospirae* (9.9% s.d. 4.4%). Candidate Phyla Radiation (CPR) taxa were detected in all samples, with the most abundant phyla being OP3 and OD1, that together accounted for 13.7% s.d. 6.5% of the community. The microbial composition was rather stable among samples, with the exception of storage tank 2017 and bathtub 2016. Spring and hydra instead displayed a remarkably stable community between the two time points. In addition, Shannon \alpha diversity values were comparable between the same sites sampled in the two time points (Fig. 1b); samples collected in different years from spring and hydra had a similar value, with a markedly higher diversity for the spring compared to hydra. Conversely, the storage tank and the bathtub showed a highly fluctuating diversity between sampling points. In beta diversity analyses (Fig. 1c) spring and hydra samples from different years tightly clustered together, while the two other sampling points were clearly separated. It can be noted that the 2016 storage tank sample had a very similar community composition compared to the spring samples.

### Shotgun metagenomic sequencing reveals a high proportion of undescribed taxa

**Fig. 2:**
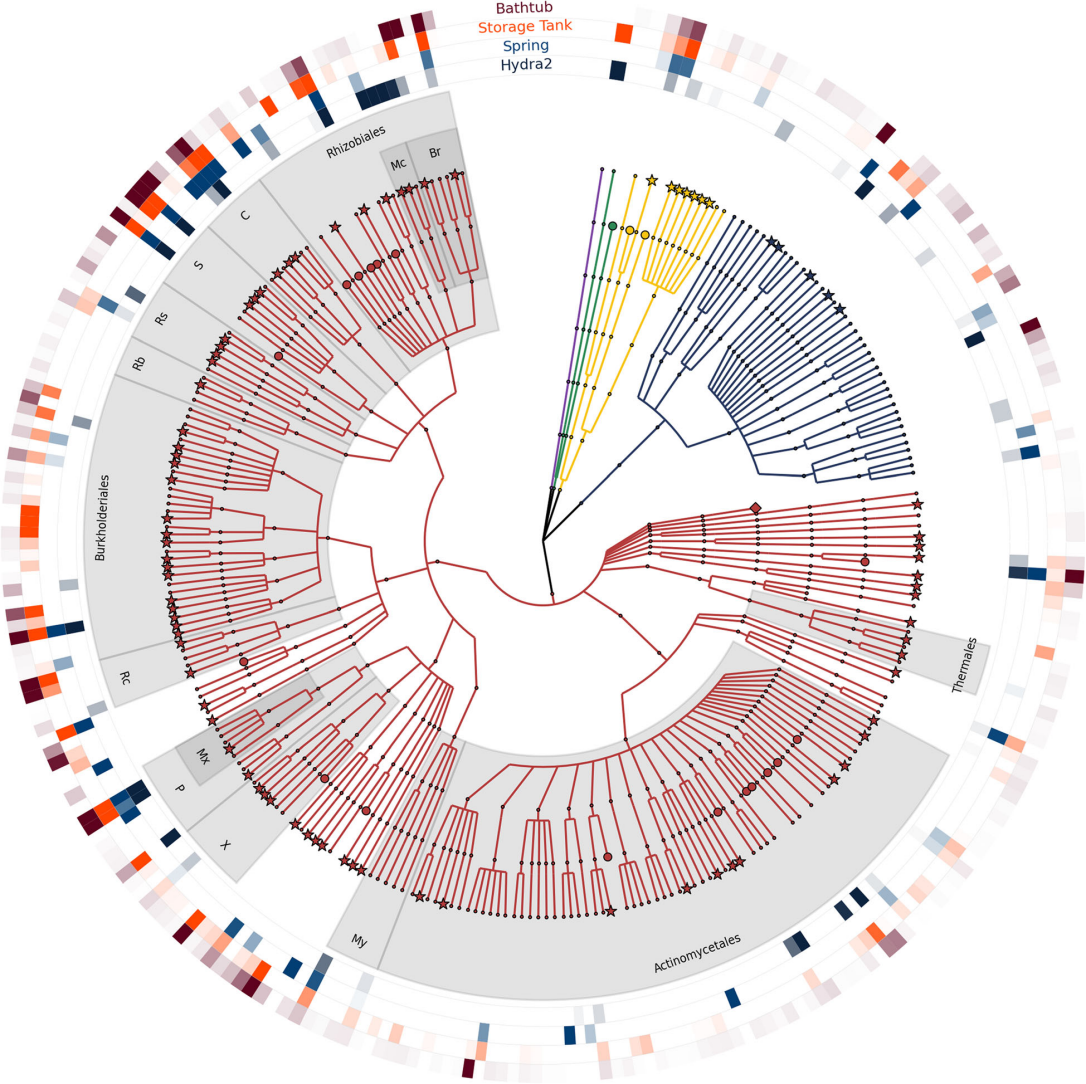
Taxonomic tree of taxa detected in Comano Terme water by shotgun metagenomic sequencing. The external rings represent microbiome composition at the four sampling sites, with maximum color intensity corresponding to a relative abundance > 1%. Branches are color-coded according to the domain of life: yellow, *Archaea*; red, *Bacteria*; green, *Eukaryota*; purple, viroids; blue, viruses. Shapes of the leaves of branches are coded as follows: small dots, described species; stars, unclassified species; circles, unclassified genera; diamonds, unclassified orders. Taxa are abbreviated as follows: C, Caulobacterales; S, Sphingomonadales; P, Pseudomonadales; Rs, Rhodospirillales; Rb, Rhodobacterales; X, Xanthomonadales; My, Myxococcales; Rc, Rhodocyclales; Mc, Methylocystaceae; Br, Bradyrhizobiaceae; and Mx, Moraxellaceae

After quality filtering, the four libraries consisted of 46.624.517 s.d. 21.730.638 read pairs, for a total of 372.996.136 reads (Table 2). The four samples differed markedly in taxonomic composition and community structure (Fig. 2, Additional file 3: Figure S1). Relative abundances of the taxa inferred by MetaPhlAn2 show that *Bacteria* have the highest abundance 78.4% (196 taxa), followed by viruses with 15.6% (39), *Archaea* with 4.8% (12), *Eukarya* with 0.8% (2), and viroids with 0.4% (1). A total of 108 out of 250 (43.2%) unclassified taxa were identified, mainly at the species (84/108, 77.8%) and genus (23/108, 21.3%) levels. In only one case an unclassified order was found, belonging to the class *Opitutae*. Consistently with 16S profiling, *Proteobacteria* dominated the water microbiome in all sampling sites, although in hydra and spring, the dominance was less pronounced (68.9% and 72.2% versus 84.4% and 84.5% in storage tank and bathtub, respectively).

**Table 2:** Main shotgun metagenomic sequencing statistics for each sampling point

	Hydra	Spring	Storage tank	Bathtub
# read pairs	29.853.009	34.542.495	44.122.903	77.979.661
# contigs (> 1000)	36.646	17.797	20.035	44.921
Longest	194.557	113.187	23.989	14.321
Average	2.768	2.125	1.764	1.973
N50	3.558	2.103	1.722	1.934
Total base pairs (assembled contigs)	101.428.889	37.810.240	35.332.755	88.647.607
GC %	55.02	58.42	57.56	56.55
% reads mapping	40.51	14.68	8.67	13.12

The core microbiome included the orders *Sphingomonadales*, *Rhizobiales*, and *Caulobacterales*, and the families *Bradyrhizobiaceae* and *Moraxellaceae*. The majority of core taxa, as for instance *Actinomycetales* and *Burkholderiales*, were found at a different relative abundance in the four sampling points*.* Viruses represent the second most abundant source of DNA and are rather homogeneously distributed between all samples. The family *Methylocystaceae* (*alpha*-*Proteobacteria*) was among the major components of the hydra microbiome (39.5%), whereas in spring, storage tank, and bathtub its abundance was much lower (0.4%, 0.03%, and 0.1% respectively) (Additional file 3: Figure S1a).

### Isolate genomes and MAGs cover a wide range of taxa and abundance classes

To select the isolate genomes to be sequenced, the culture collection was pre-screened by amplifying and sequencing the 16S rRNA gene from single colonies and by clustering the isolates sharing an identity above 99%. For each cluster, a representative isolate was chosen. Whole genomes of 77 out of 181 isolates were sequenced. After assembly and quality checks, five genomes were excluded. A summary of genome assembly statistics is presented in Additional file 4: Table S3 and Additional file 5: Table S4. The metagenomics assembly resulted in 29.850 s.d. 13.101 contigs, summing up to > 500 Mbp (Table 1). After concatenation and redundancy removal with CD-HIT, the number of contigs was 114.739 with a total length of 255.640.704 bp. The initial automatic binning process resulted in 63 bins (MAGs), but many of them were either contaminated (probably resulting from a mixture of multiple, highly similar genomes) or shorter than 0.5 Mbp. After manual curation and quality filtering, 30 bins (accounting for 58.141.697 bp) passed the thresholds set by MIMAG. Ten out of 30 MAGs had > 90% completion and < 5% contamination, while 12 out of 30 MAGs had > 18 tRNAs, and in 2 MAGs all 3 ribosomal RNA genes were found (Additional file 4: Table S3). According to the single copy gene classifier implemented in Anvi’o, 21 bins belonged to a bacterial phylum, 3 bins were classified as Archaea, and 6 bins were assigned to the CPR super-phylum [12].

**Fig. 3:**
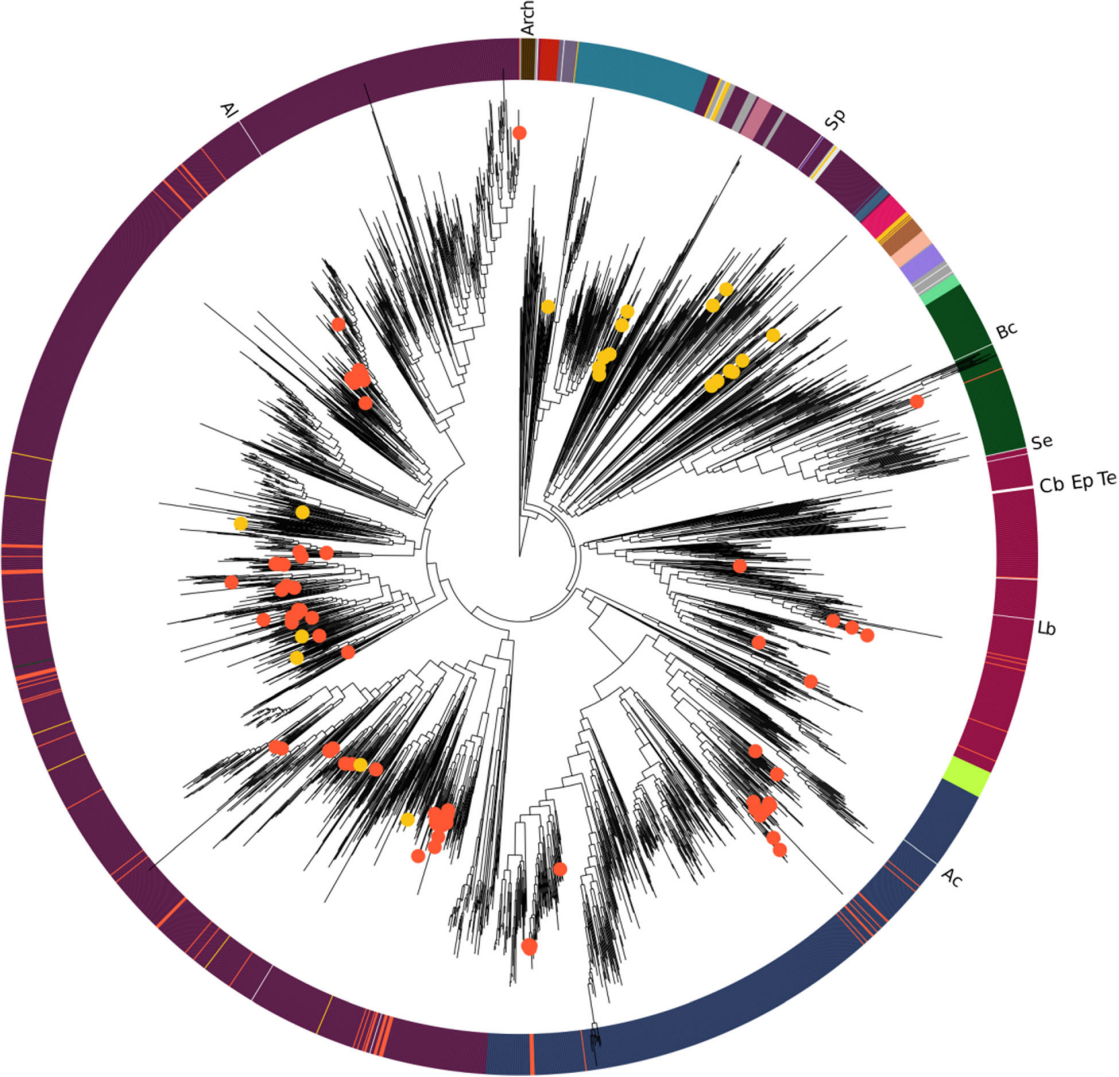
Phylogenetic position of bacterial isolates and MAGs on the bacterial tree of life. Orange dots, isolate genomes; yellow dots, MAGs. Branches that do not contain any isolate genomes or MAGs were collapsed for clarity. Collapsed branches are reported with an abbreviation on the external ring as Arch, Archaea; Ac, Actinomycetaceae; Al, Alteromonadales; Bc, Bacteroidales; Cb, Coriobacteriales; Ep, Erysipelotrichia; Lb, Lactobacillales; Sp, Spirochaetes; Se, Selenomonadales; and Te, Tenericutes. The external ring is colored according to the phylum

The genomes of the bacterial isolates included 4 phyla and 6 classes. Thirty-nine (54.1%) genomes resulted to be unclassified at different levels: 19 at the genus level, 16 at the family level, and 4 at the order level. While MAGs covered 4 phyla and 6 classes, for 13 and 7 of them, it was possible to infer their taxonomy at the order and at the species level, respectively (Additional file 5: Table S4). Isolates and MAGs were widely distributed in the microbial tree of life (Fig. 3).

While all isolates could be taxonomically identified by PhyloPhlAn2, reaching the number of 100 aligned universal marker threshold for taxonomic placement, 9 MAGs failed to pass this threshold, probably as a consequence of their small genome size. Fragment recruitment analysis, i.e., the percentage of metagenomic reads mapping on a given MAG, revealed that 24 MAGs were reconstructed from hydra sample, including all *Archaea* and CPR. These accounted for 58.82% of the reads, while the two MAGs reconstructed mainly from the spring sample accounted for 3.38% and the four MAGs reconstructed mainly from the bathtub sample accounted for 9.53% of the reads, respectively.

**Fig. 4:**
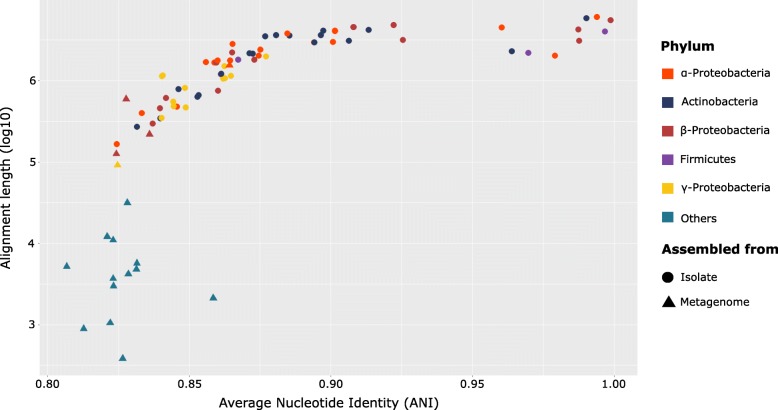
Average nucleotide identity (ANI) values (*x*-axis) plotted against log_10_-transformed alignment length (*y*-axis). Each point indicates the best ANI hit between a given isolate genome (dots) or MAG (triangles) and its closest relative according to the database implemented in PhyloPhlAn2. Dots are color-coded according to the phylum. The threshold used for species and genus delineation is 95% and 85%, respectively

ANI values calculated between each genome isolated (or assembled) from the spring water and their closest relative in the set of publicly available genomes [19], as inferred by PhyloPhlAn2, showed that 10 genomes belonged to known bacterial species (> 95% ANI over an alignment length > 1Mbp) (Fig. 4), 36 genomes (including 1 MAG) had ANI values ranging 85–92.5% over an alignment length of > 1 Mbp, and could be assigned as undescribed species belonging to known genera. The remaining 55 genomes had low ANI values (< 85%) and/or alignment length (< 1 Mbp). Few isolate genomes and most MAGs belonged to the latter group, with an alignment length ranging from few hundreds to few thousands base pairs and ANI values comprised between 80% and 85% (Fig. 4). In four cases, a MAG clustered within the same clade of an isolate with a relatively narrow phylogenetic distance. For those pairs, ANI values suggest that they may belong to the same family. Specifically, Bin_15_1 showed an ANI value of 72.5% with the isolate L1A1; Bin_9_1 had ANI value of 71.3% with 3R27C2-B; Bin_43, 70.8% with the isolate L1B and Bin_16_1 has an ANI of 68% with isolate 2PA. No MAGs were found to have a species or genus level relationship (ANI value > 95% or > 85%, respectively) with any of the isolates (Fig. 3).

### Metagenomic annotation reveals functional differentiation among the four sampling sites

The analysis of the metabolic potential encoded in the metagenomic dataset, as inferred by HUMAnN2, revealed 176 core functions detected in all samples out of 314 (Additional file 6: Figure S2). Functions detected uniquely in hydra, spring, storage tank, and bathtub samples were 4, 3, 18, and 17, respectively (Additional file 6: Figure S2). The most functionally similar samples were storage tank and bathtub (30 shared functions), while the most dissimilar samples were hydra and spring (no shared functions).

**Fig. 5:**
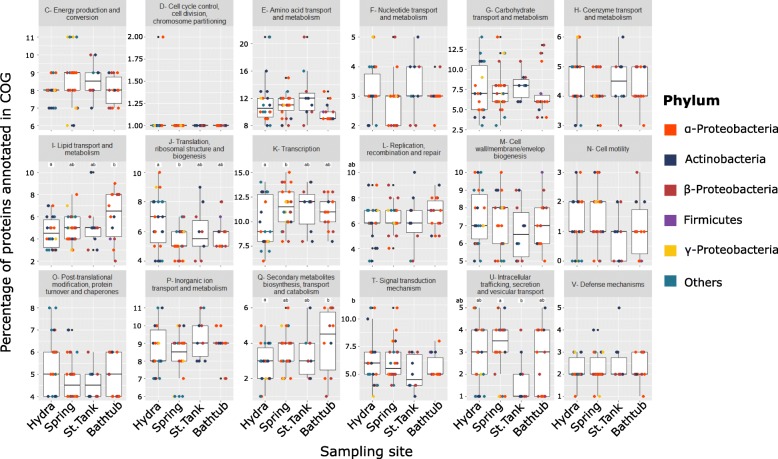
Percentages of proteins annotated within each COG category for the four sampling sites. Dots are colored according to the phylum. Differences between the distributions of percentages in two or more sites were inferred by two-way ANOVA followed by Tukey post hoc test. Significant differences are shown as “a,” “ab,” or “b” categories marked with the same letter are not significantly different

Functional features were also investigated among the genomes of isolates using EggNOG-mapper. Proteins were classified according to COG categories (Fig. 5), and in the following cases site-specific differences were found: genes involved in lipid transport and metabolism were significantly enriched in the genomes deriving from the bathtub compared to hydra sample. Significant differences were also found in genes involved in transcription and translation between hydra and spring samples. Other significant differences were found in COG category Q (secondary metabolites biosynthesis, transport, and catabolism), which were enriched in bathtub compared to hydra, and in COG category U (intracellular trafficking, secretion, and vesicular transport), which were more abundant in spring compared to storage tank (Fig. 5).

The total number of gene families detected by EggNOG-mapper including both isolate genomes and MAGs was 47,092, with an average of 2.886.49 s.d. 1.423.71 gene families per genome. The largest number of gene families were detected in genome 4NA327C10 (5.683), classified as *Burkholderia* spp., while the lowest was in the MAG Bin_46_1 (407), an undescribed bacterium. Globally, MAGs had a lower number of detected gene families, with 14 MAGs out of 30 containing less than 1000 gene families. The MAG with the highest number of gene families detected was Bin_11_1 (2710). 16.431 gene families occurred in only 1 genome while 39.458 gene families were found in less than 10 genomes.

**Fig. 6:**
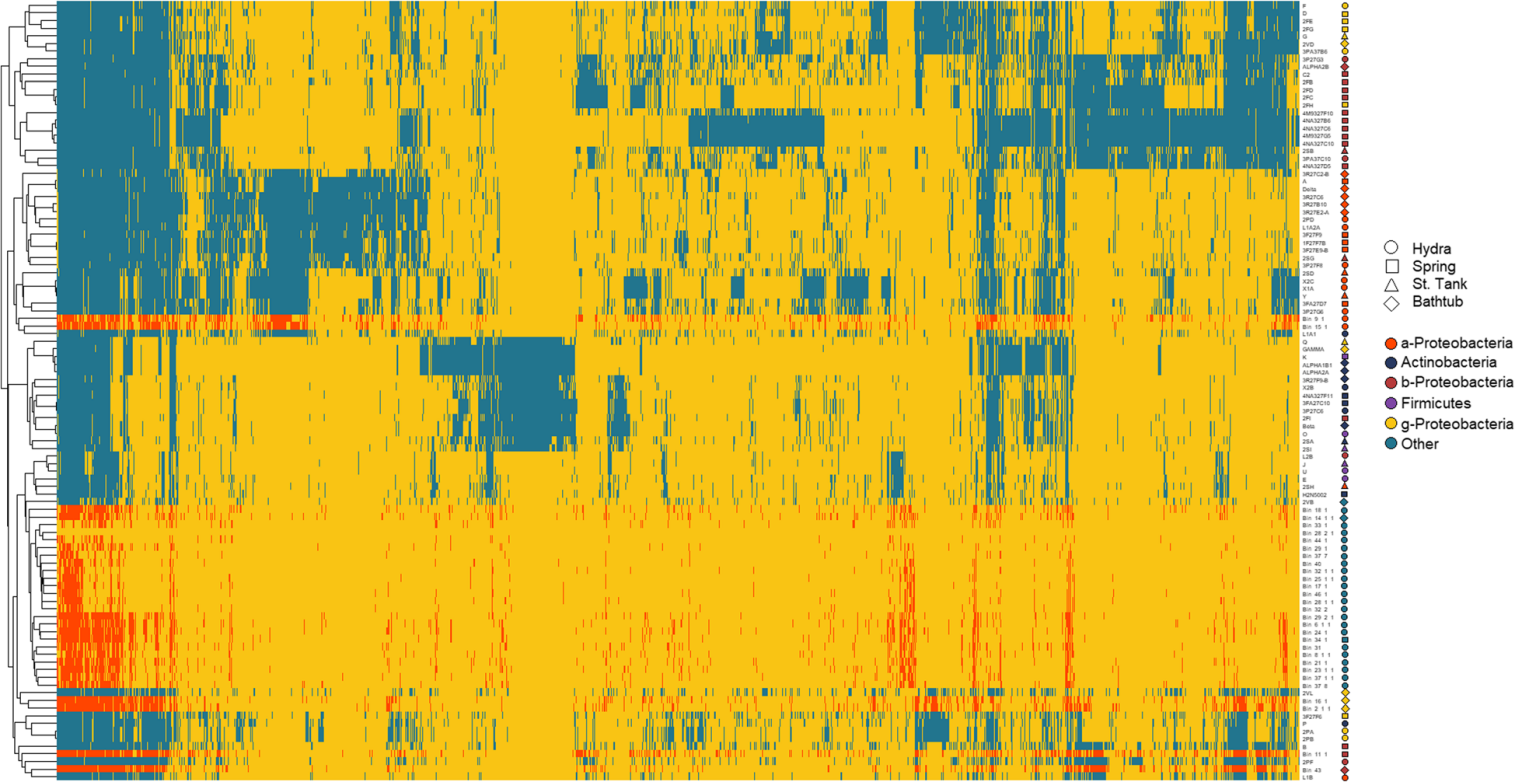
Binary heatmap displaying the gene families present in at least 10 genomes. Gene families (columns) were hierarchically clustered according to their presence in the genomes (blue) or in the MAGs (red). Site of isolation (shapes) and taxonomic position (color) are indicated for each isolate genome or MAG on the right

Genomes were clustered according to the presence/absence of 7.634 gene families that were found in at least 10 genomes (Fig. 6). Thirty-six gene families displayed a correlation between their presence (or absence) and sampling site (Additional file 7: Table S5, Additional file 8: Figure S3); 28 gene families were significantly absent in isolate genomes (or MAGs) derived from hydra, 2 of them were significantly more present in genomes derived from spring, 5 of them were significantly more present in storage tank, and 1 in bathtub. However, in the latter two cases, the Benjamini-Hochberg correction for false discovery rate showed that the correlation was not significant (Additional file 7: Table S5).

## Discussion

The microbiome of a thermal spring (Comano Terme, Italy) was investigated using both metagenomic and culturomic approaches, leading to the isolation in pure culture of 72 bacterial strains and the assembly of 30 MAGs from the metagenomic dataset. Many isolates are likely derived from the fraction of the microbiome with low abundance whereas MAGs provide an overview of the most abundant, unculturable fraction of the microbiome [14]. The reason for this is intrinsic to the properties of the microbial community assembly, usually dominated by very few species; therefore, high throughput cultivation would likely retrieve many species which in the original community have low abundance [14]. MAGs instead necessarily derive from abundant species because a genome must be well covered to be assembled, implying that many cells must be present in the original sample. In fact, one of the MAGs (Bin_15_1) was classified as *Methylocystis* sp. and was assembled mainly from hydra. This bacterium might be the same one found at high abundance in hydra using MetaPhlAn2. Remarkably, nearly 40% of the hydra shotgun reads mapping on taxonomically informative marker genes were from this taxon. The analysis of 16S amplicon sequencing data supported this statement, as *Methylocystis* sp. was among the most abundant taxon found in hydra. On the other hand, MAG Bin_16_1, classified as *Methylobacter* sp., was assembled mainly from the bathtub metagenome, although it was well covered also in storage tank and spring, but not in hydra. Metagenomic shotgun data analyzed with MetaPhlAn2 revealed that the order *Methylococcales* (which includes the *Methylobacter* genus) was absent in hydra, and had a relative abundance ranging 0.5–1.4% in the metagenomes of the other sampling sites.

Interestingly, the taxonomic profiles of hydra and spring, as inferred not only by metagenomic analyses but also from the genomes of the isolates, recall the ones typical of soil environments [20]. Moreover, some taxa (for instance, *Bradyrhizobiaceae* and *Rhizobiales*) are known to be associated with the rhizosphere. These bacteria most probably derive from the water percolating through the overlying soil column, which carries both organic matter and microorganisms to the saturated zone [21]. Our sampling plan included two natural environments (hydra and spring), and two artificial ones (storage tank and bathtub). Hydra was the most peculiar one; the contigs obtained from this assembly did not differ much in sequence composition compared to the other environments but had a markedly different coverage profile (Additional file 9: Figure S4). This is in agreement with previous findings on groundwater microbial communities, which are known to be distinct from the ones of corresponding surface streams [5]. Most MAGs were assembled from this sampling point and belonged to phyla typically recalcitrant to cultivation. Pristine aquifers are characterized by relatively low diversity and high stability [3, 5]. Our data confirms both features, as hydra was the site with the lowest number of detected taxa (80), and the taxonomic composition inferred by 16S amplicon sequencing was nearly identical in both time points sampled. Many isolates and most MAGs were classified as new genera or higher taxonomic ranks, probably due to the peculiarity of this groundwater-fed spring environment. An increased rate of speciation is indeed recognized to be typical of highly adapted populations living in very stable environments [22, 23].

Core functions as inferred by HUMAnN2 highlighted the importance of lifecycle, biosynthesis and energy metabolism. Catabolic pathways found in the core functions included glycolysis and degradation of plant-derived compounds. Methanogenesis appears as one of the unique functions of hydra, whereas methanotrophic functions were found in all samples, which is supported by the presence of *Methylocystaceae* in the core metagenome. Methanogenesis and methanotrophy have been studied in other groundwater ecosystems [5, 24, 25]. However, most of the current knowledge about methane source-sink dynamics in aquatic environments derives from surface waters [26–28]. In such environments, methanogenesis is mainly performed by Archaea. This process occurs in anaerobic microniches such as deep sediments in river basins or lakes, and consists mainly in hydrogenotrophic methanogenesis [26, 29]. In our setting, methanogenesis seems to be a specific feature of the groundwater sample (hydra). Several archaeal taxa were detected from the groundwater metagenome: three MAGs were classified as Archaea, and MetaPhlAn2 analysis revealed that an unclassified genus of the thermophilic family methanocaldococcaceae (Euryarchaeota) was detectable only in the hydra sample. This family is known to perform hydrogenotrophic methanogenesis. Functional analysis revealed that in hydra, the methanogenesis mainly relies on the H_2_/CO_2_ pathway (Additional file 6: Figure S2) [29].

Notably, an opposite trend for COG categories J and K was found in hydra compared to spring. Gene families involved in translation, ribosomal structure, and biogenesis (COG category J) were significantly overrepresented in hydra, whereas transcription (COG category K) were underrepresented. Although this aspect deserves further investigation, it is consistent with the fact that groundwater microorganisms (defined as “unusual” from Brown et al. 2015) genome structure lacks primary biosynthetic pathways, contain introns and miss specific ribosomal proteins [12].

## Conclusions

Our results confirm that groundwater environments host highly adapted, stable microbial communities. We showed the occurrence of a high functional partitioning where methane production and consumption are spatially separated. Current knowledge on aquatic microbiomes is essentially based on surface (sea, lakes, streams) or human-associated (drinking water, sewage) environments. This study should constitute a basis for future investigations of the groundwater microbiome.

## Materials and methods

### Spring and sampling points

The thermal spring of Comano Terme (46°02′31.0′′ N; 289 10°53′09.7′′ E) has a chemical composition dominated by bicarbonate, calcium, and magnesium salts. It displays a constant water flow of 3 l/s and a constant temperature of 27 °C during the year. The water is indicated for the treatment of inflammatory skin conditions (psoriasis and atopic dermatitis) [30, 31]. The spring feeds the nearby Comano Terme spa through a piping system. In addition to the natural spring, the basin of thermal water is accessible through an artificial well named hydra (46°02′32.9′′ N; 10°52′58.3′′ E) that is connected with the piping system of the spa and used as an additional water supply in case of necessity, typically during the summer season. The system also comprises a storage tank, where the water is heated at 37 °C before to be delivered to the bathtubs used for balneotherapy. In this system, we identified four sampling sites: the hydra well, the spring (Antica Fonte), the storage tank, and a bathtub.

All samples were collected in sterile bottles. Immersion samples (spring and storage tank) were taken using singularly packed sterile bottles and handled with sterile forceps. For samples taken from taps (hydra well and treatment bathtub), the tap was sterilized by flame and the water was let flow for 1 min before sampling. Quantification of the total bacterial load was performed on 3 samples of 1 l following the procedure described by Paul et al. [32]. For shotgun sequencing and 16S amplicon sequencing, 4 l were sampled. Water bottles were kept at air temperature (about 20 °C) during transportation to the laboratory and were processed within 6 h.

### Strain isolation and identification

Water samples of 100 ml were concentrated by filtration using 0.22 μm Pall Supor filters (Pall Corporation, Port Washington, NY, USA). Filters were then placed on standard Petri dishes poured with four different growth media. In order to be able to culture the highest number of bacterial species, three common non-selective media (nutrient agar, tryptic soy agar, plate count agar) and one medium for enumeration of bacteria from potable water (Reasoner’s 2A agar) [33–35] were used for bacterial isolation. Growth media were prepared using filtered water from Comano Terme spring in order to maintain the native chemical composition of this environment.

To maximize the number of cultivable isolates, a large-scale culturing approach was set up following the protocol of Connon and Giovannoni [36]. Based on the microbial load evaluation, 2 μl of Comano Terme water (1–10 cells) were inoculated into 2 ml of growth medium in 96-well plates with square walls and round bottom; edges of the plate were filled with water to prevent edge effect. Four different media were used for liquid cultures, two media for enumeration of bacteria in water (Reasoner’s 2A agar and King’s B [37]), one non selective medium (nutrient broth), and one low**-**nutrient medium (M9 minimal medium). Each medium was prepared in five versions: standard, two pH variants (pH 5.5 and pH 8), one variant with addition or subtraction of carbon source (glucose, soluble starch, or both), and one amino acid variant (with or without yeast extract). Plates were replicated and incubated at 27 °C and 37 °C in both aerobic and anaerobic conditions. Anaerobic cultures were grown in a Ruskinn Concept 400 Anaerobic Workstation (Ruskinn Technology Ltd., Bridgend, UK) and maintained during incubation using GENBAG, Anaerobic Atmosphere Generator (bioMérieux, Marcy-l’Étoile, France). Evaluation of growth was performed for each well measuring O.D. 600 values with a TECAN Infinite m200 PRO microplate reader (Tecan, Männedorf, Switzerland). Wells displaying growth were then tested for purity performing two rounds of streaking on R2A plates. After 7 days of incubation at 27 °C, glycerol stocks were prepared. The 16S rRNA gene of each isolate was amplified by PCR for taxonomic identification. DNA was extracted using ReliaPrep gDNA extraction kit (Promega, Milan, Italy) and amplified using standard primers 8F-1492R [38]. Amplicons were sequenced using Sanger sequencing service by Eurofins (Eurofins Scientific, Luxembourg). Sequences with length > 400 bp and an average Phred score of > 25 were used for taxonomic identification of isolates using RDP classifier [39]. Assigned taxonomies were accepted only at estimated confidence > 95%.

### 16S amplicon sequencing

Two samples of 4 l were collected from each sampling point (3 replicates) in February 2016 and in August 2017. Total DNA was extracted using RapidWater DNA extraction kit (MoBio, Carlsbad CA, USA) with minor modifications: at step 5 of the protocol, the PowerWater Beat Tube were heated at 65 °C for 10 min and mechanical cell lysis was extended to 10 min for all samples. All other steps were performed following the manufacturer’s instructions. DNA extraction of 2017 samples was performed on triplicates and extraction yield remained consistent for all samples (data not shown). The V4 hypervariable region of the 16S rRNA gene was amplified by PCR with the 5PRIME HotMasterMix (Quanta BIO, Beverly, MA, USA). The length of the amplicons was 253 base pairs. Negative controls were included during sampling and main wet-lab steps. PCR blanks, DNA extraction blanks and DNA extractions from unused filters were prepared. Amplicons concentration, size range, and purity were measured using Agilent high sensitivity (HS)DNA kit on the Bioanalyzer 2100 (Agilent Technologies Italia S.p.A, Milano, Italy). Based on the molarity estimated using Bioanalyzer, each PCR product was diluted before pooling. The final pool was purified using the Agencourt AMPure XP DNA purification kit, following manufacturer’s instructions. Amplicons were sequenced on an Illumina MiSeq platform with 2 × 300 bp paired-end protocol.

The fastq files were quality checked using FastQC [40], and initial sequence analysis was performed with QIIME1 [41]; after demultiplexing and primer removal, reads were joined using default parameters. Joined reads were then filtered by average Phred quality score > 25 and a minimum length of 250 nucleotides; reads longer than 255 nucleotides were trimmed. Operational taxonomic units (OTUs) were built at 97% sequence identity with uclust [42]. Taxonomic assignment was performed with RDP classifier [39], using the Greengenes [43] reference dataset (release gg 13 5). Diversity analyses were performed using QIIME on a rarefied dataset to prevent statistical artifacts due to different sequencing depths. Alpha diversity was measured with Shannon metrics. Beta diversity measures were measured on unrarefied dataset calculating weighted UniFrac metrics and displayed through 2D PCoA [44].

### Shotgun sequencing

Samples of 4 l were filtered using 0.22 μm Pall Supor filters (Pall Corporation, Port Washington, NY, USA). DNA extraction from filter was carried out using the RapidWater DNA extraction kit (MoBio, Carlsbad, CA, USA). Sequencing libraries were prepared using Nextera XT DNA Library Preparation Kit (Illumina, San Diego, CA, USA) following the manufacturer instructions, and quality checked using Caliper LabChip GX. Sequencing was performed on an Illumina HiSeq 2500 platform (Illumina, San Diego, CA, USA) with 100 bp paired-ends and an insert size of 250 bp. Raw sequencing reads were quality checked using fastq-mcf [45], and reads derived from human contamination were removed after mapping on the human genome (version hg19) using bowtie2 [46]. Adaptors and residual synthetic constructs were removed first mapping the reads on the genome sequence of the phage Phy174, and then using trim galore [47].

Microbiome profiling based on a database of taxon-specific marker genes was performed using MetaPhlAn2 [48]. The output of MetaPhlAn2 analyses was plotted using GraPhlAn [49].

### Metagenome-assembled genomes (MAGs)

Each of the four samples was assembled separately using megahit [50], with the preset–meta-sensitive and setting the minimum contig length to 1000 bp. The assembled files were then concatenated, and redundant contigs were removed using CD-HIT [51], with 100% coverage and 100% id. The reads derived from the sequencing libraries were mapped on the resulting co-assembly. Downstream analysis was performed using Anvi’o v2.4.0 [52] and included the following steps: (i) taxonomic and functional annotation of the contigs; (ii) profiling the mapping files of single libraries on the co-assembly, to assess the coverage of each contig from each library; (iii) binning contigs into putative MAGs, based on the tetramer composition and differential coverage using CONCOCT [53], as implemented in the Anvi’o pipeline. A further quality check of MAGs relied on the presence of a set of single-copy genes [54]; the proportion of single-copy genes present in each MAG is considered an indicator of genome completedness, while the presence of multiple copies of the same single-copy gene (redundancy) is an indicator of possible contamination. Bins of contigs were manually curated to minimize the contamination by removing contigs (or groups of contigs) that showed markedly different coverage profile and/or tetrameric signature. The annotation of both MAGs and isolate genomes was performed using Prokka version 1.12 [55]. MAGs were classified into high and medium quality, following the guidelines for minimum information about metagenome-assembled genome (MIMAG) [56]. In brief, high-quality genomes had > 90% completeness, N50 > 10 Kb, < 5% contamination, < 500 contigs, encoded all three ribosomal RNA genes and at least 18 tRNAs; medium-quality genomes had > 50% completeness and < 10% redundancy. In addition, a score was calculated as *S* = completeness percentage − 5 × (redundancy percentage), as in Parks et al. [57]. For subsequent analyses, only high- and medium-quality genomes with an *S* score > 50 were considered. A dedicated pipeline for the assignment of bins to the candidate phyla radiation (CPR) super-phylum was setup, and the corresponding score was calculated using the CPR-specific single copy genes.

### Genome sequencing and assembly

16S rRNA sequences obtained from the isolated strains were clustered using CD-HIT with a threshold of 99%. One representative isolate was selected from each cluster for genome sequencing. DNA extraction and preparation of sequencing libraries was performed as described in the shotgun sequencing section.

The quality of sequencing data was checked with MultiQC [58] and successively processed with Trim Galore [36] for quality and adapter trimming, with a quality threshold set at Phred 25. The filtered reads were de novo assembled using SPAdes v. 3.11.1 [59], using default settings and specifying kmer sizes -k 23,43,63,83,103,113. Quality of the assembled reads was verified using Quast [60]. Taxonomic placement was performed using PhyloPhlAn2 [61]; using the standard phylophlan database with 400 universal marker genes, a 2.0. Supermatrix_aa.cf. configuration, the option “—diversity” set to “high” and the “—fast” option on. The genome of the closest relative to each isolate was used to calculate the average nucleotide identity (ANI), using the software pyani [62].

### Functional potential annotation of isolate genomes and MAGs

To get an insight on the functional features encoded in the metagenome, raw unassembled reads were annotated using HUMAnN2 [63], mapping the reads on the UniRef90 database [64], and parsing the “pathabundance” output. In addition, amino acidic sequences predicted by Prokka were used as input to EggNOG-mapper to infer functional features based on orthology prediction [65]. The tabular output file of eggnog mapper includes the annotations for each protein based on gene families, KEGG maps, and COG categories. This file was imported in R [66], to get a graphical representation of the abundance of proteins as a function of COG categories and gene family distribution. The relative differential abundance of proteins assigned on a given COG category among genomes isolated in each of the sampling sites was determined through one-way ANOVA followed by the Tukey post hoc test to point the significant differences among the four sampling sites. Significant correlation between gene family presence/absence and the site of sampling was assessed using Scoary [67].

## Additional files

Additional file 1: **Table S1.** Assembly statistics and taxonomy for each genome sequenced. (XLSX 18 kb)

Additional file 2: **Table S2.** Number of 16S reads for each sampling site in each year. (XLSX 8 kb)

Additional file 3: **Figure S1a.** Rank abundance plots of the families detected using MetaPhlAn2 in the four metagenomes. *y*-axis, abundances (expressed as percentage of the reads mapping on taxonomically informative marker genes); *x*-axis, families detected. (PPTX 471 kb)

Additional file 4: **Table S3.** Statistics regarding NGS output and bioinformatic pipeline used for assembling isolate genomes. (XLSX 21 kb)

Additional file 5: **Table S4.** Statistics regarding MAGs taxonomy and genome quality as defined in Parks et al. (XLSX 22 kb)

Additional file 6: **Figure S2.** Venn diagram showing the distribution of functions in the four metagenomes. Each ellipse corresponds to the metagenome of a site; the overlapping areas represents the functions that are either shared among- or unique to- each site. Unique functions are explicitly annotated. (PPTX 988 kb)

Additional file 7: **Table S5.** Gene families found to be significantly under- or overrepresented in the four sites genomes. (XLSX 12 kb)

Additional file 8: **Figure S3.** Presence (blue)/absence (yellow) heatmap displaying only the gene families found to be significantly over- (or under-)represented in genomes deriving from one of the four sampling sites. (PPTX 1073 kb)

Additional file 9: **Figure S4.** Anvi’O plots displaying the clustering patterns of assembled contigs along with their coverage by each metagenome. Leftmost graph shows the contigs clustered only by sequence composition (i.e., tetrameric signature); rightmost graph clusters the contigs only by their differential coverage; middle graph, combination of differential coverage and tetrameric composition. (PPTX 829 kb)

## Abbreviations

ANI: Average nucleotide identity
CPR: Candidate Phyla Radiation
MAG: Metagenome-assembled genome
OTU: Operative taxonomic unit
