# Supplementary material for: Genomic and metagenomic insights into the microbial community of a thermal spring
Source: Microbiome. 2019 Jan 23;7:8. doi: 10.1186/s40168-019-0625-6 (PMC6343286; doi:10.1186/s40168-019-0625-6)
Supplement: Supplementary file 3 — Figure S1a. Rank abundance plots of the families detected using MetaPhlAn2 in the four metagenomes. y-axis, abundances (expressed as percentage of the reads mapping on taxonomically informative marker genes); x-axis, families detected. (PPTX 471 kb) [file 40168_2019_625_MOESM3_ESM.pptx]

## Slide 1
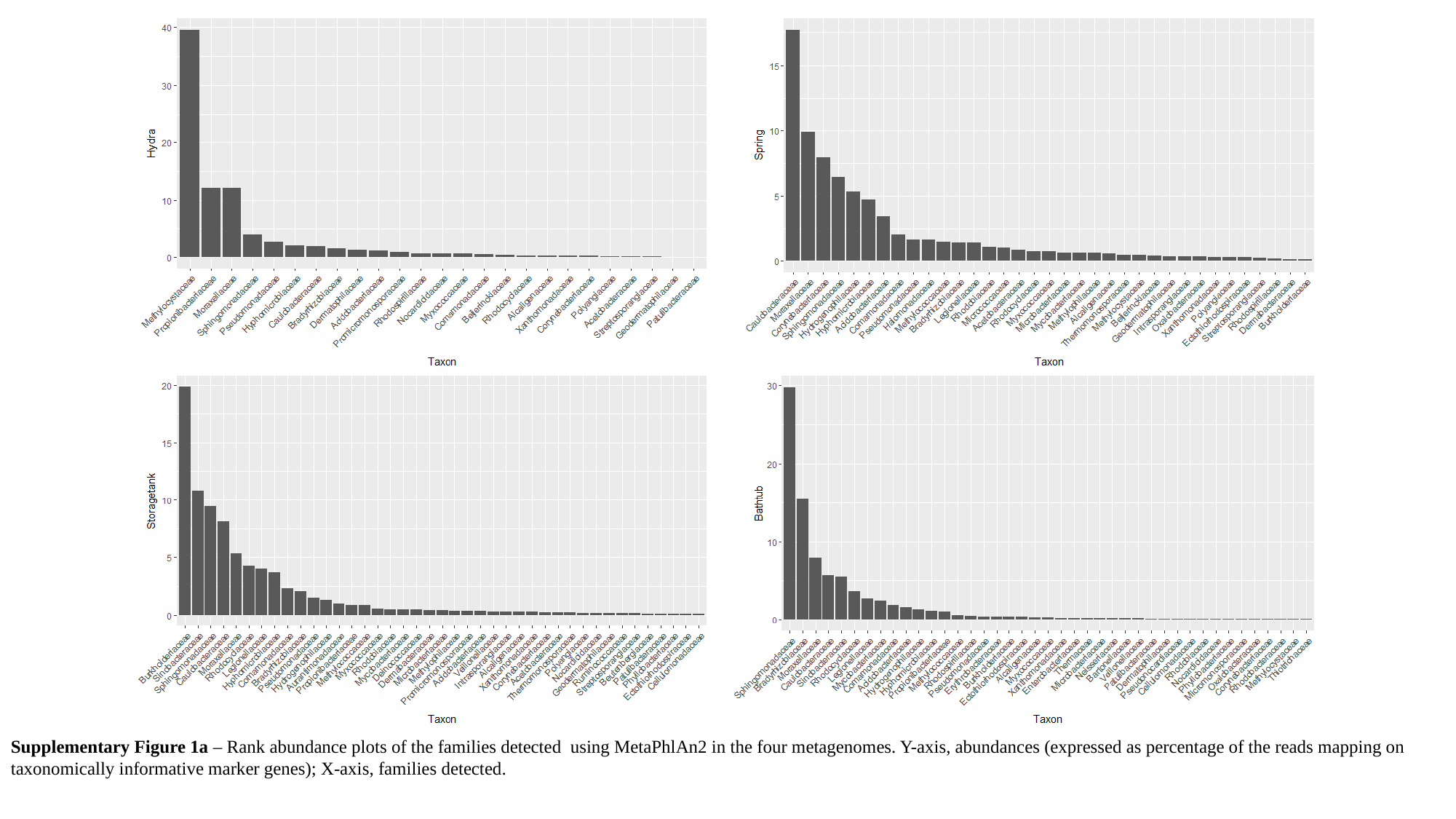

Supplementary Figure 1a – Rank abundance plots of the families detected using MetaPhlAn2 in the four metagenomes. Y-axis, abundances (expressed as percentage of the reads mapping on taxonomically informative marker genes); X-axis, families detected.

## Slide 2
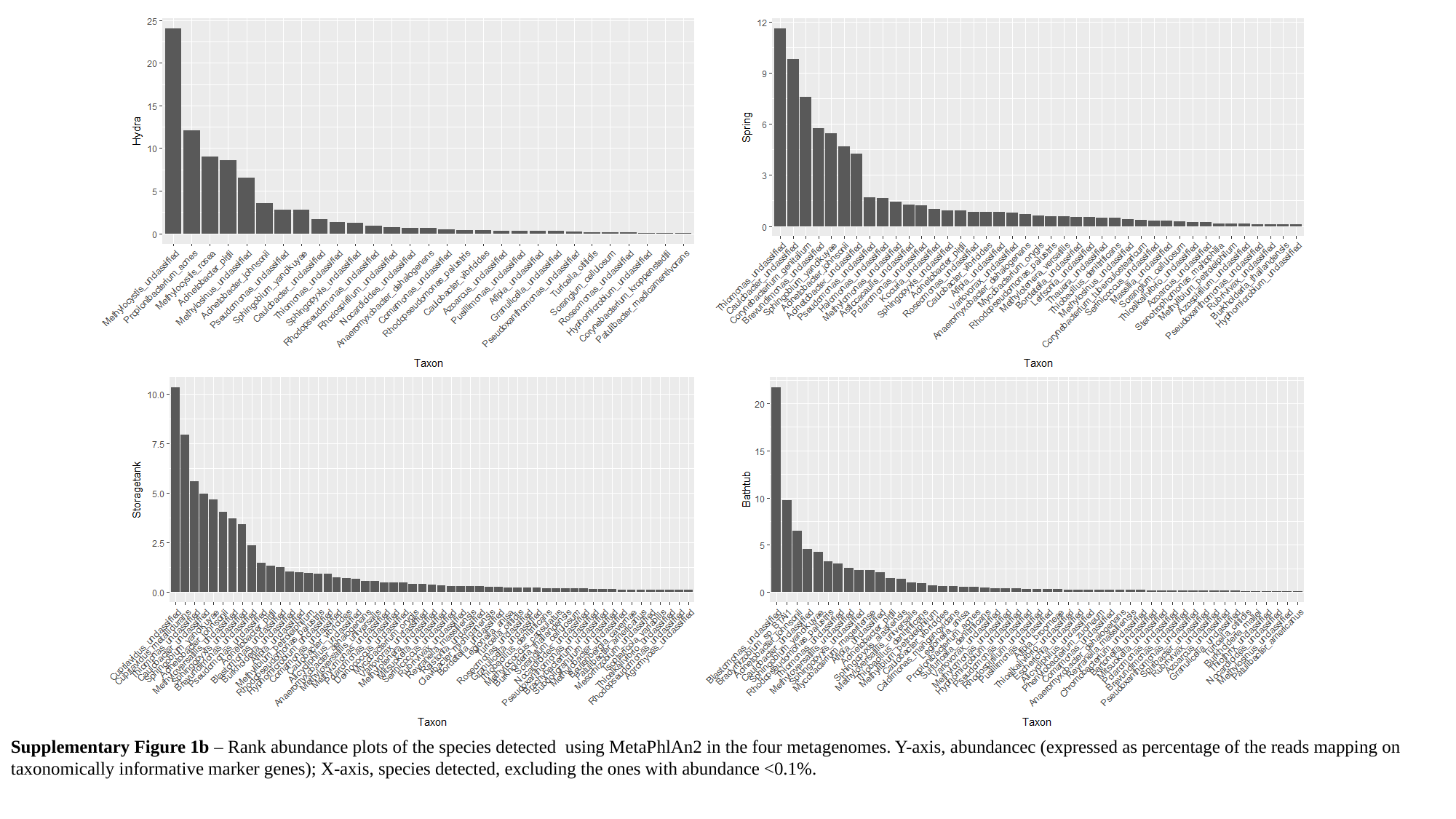

Supplementary Figure 1b – Rank abundance plots of the species detected using MetaPhlAn2 in the four metagenomes. Y-axis, abundancec (expressed as percentage of the reads mapping on taxonomically informative marker genes); X-axis, species detected, excluding the ones with abundance <0.1%.
