# Supplementary material for: Genomic and metagenomic insights into the microbial community of a thermal spring
Source: Microbiome. 2019 Jan 23;7:8. doi: 10.1186/s40168-019-0625-6 (PMC6343286; doi:10.1186/s40168-019-0625-6)
Supplement: Supplementary file 6 — Figure S2. Venn diagram showing the distribution of functions in the four metagenomes. Each ellipse corresponds to the metagenome of a site; the overlapping areas represents the functions that are either shared among- or unique to- each site. Unique functions are explicitly annotated. (PPTX 988 kb) [file 40168_2019_625_MOESM6_ESM.pptx]

## Slide 1
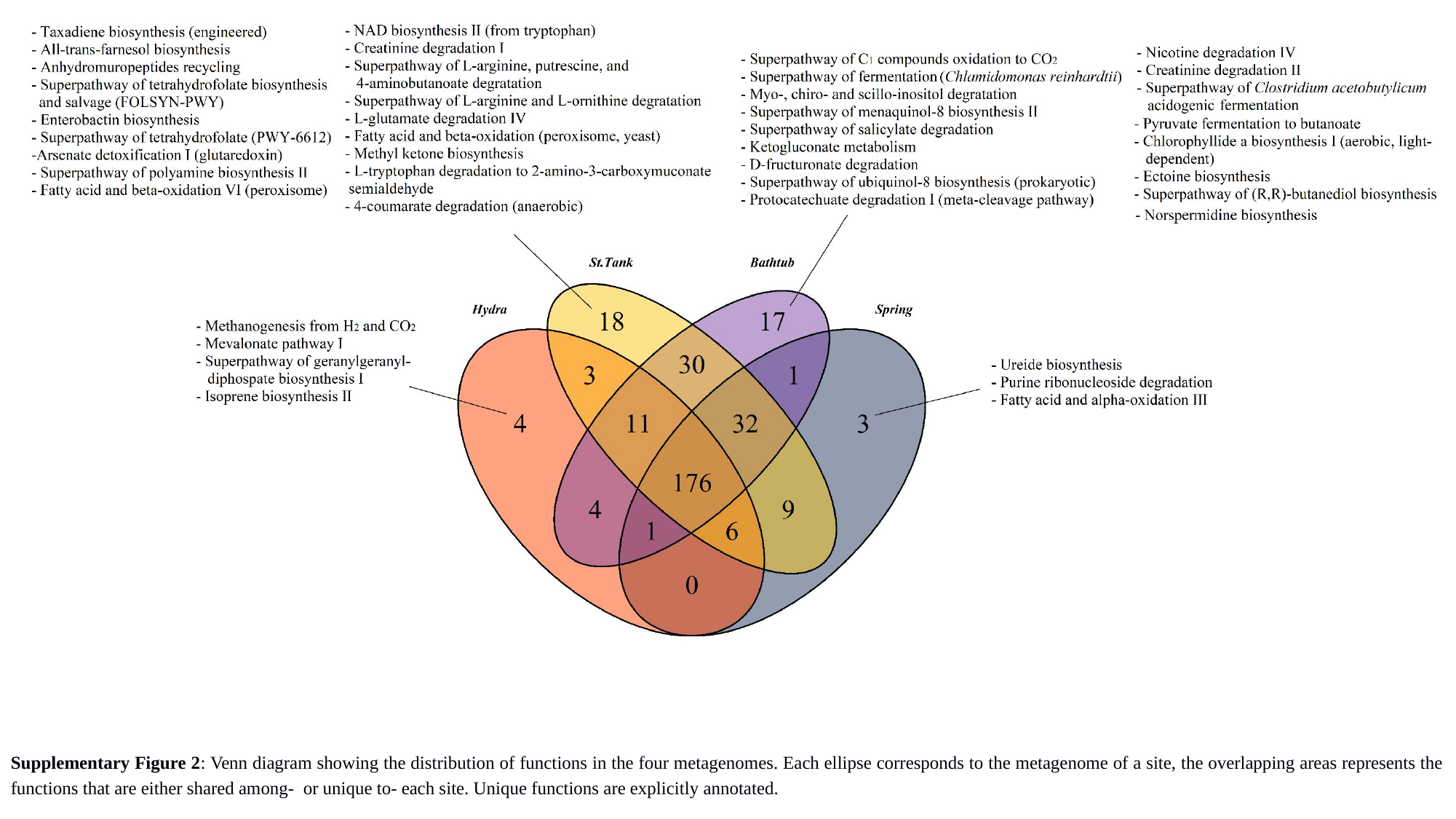

Supplementary Figure 2: Venn diagram showing the distribution of functions in the four metagenomes. Each ellipse corresponds to the metagenome of a site, the overlapping areas represents the functions that are either shared among- or unique to- each site. Unique functions are explicitly annotated.
