# Supplementary material for: Genomic and metagenomic insights into the microbial community of a thermal spring
Source: Microbiome. 2019 Jan 23;7:8. doi: 10.1186/s40168-019-0625-6 (PMC6343286; doi:10.1186/s40168-019-0625-6)
Supplement: Supplementary file 8 — Figure S3. Presence (blue)/absence (yellow) heatmap displaying only the gene families found to be significantly over- (or under-)represented in genomes deriving from one of the four sampling sites. (PPTX 1073 kb) [file 40168_2019_625_MOESM8_ESM.pptx]

## Slide 1
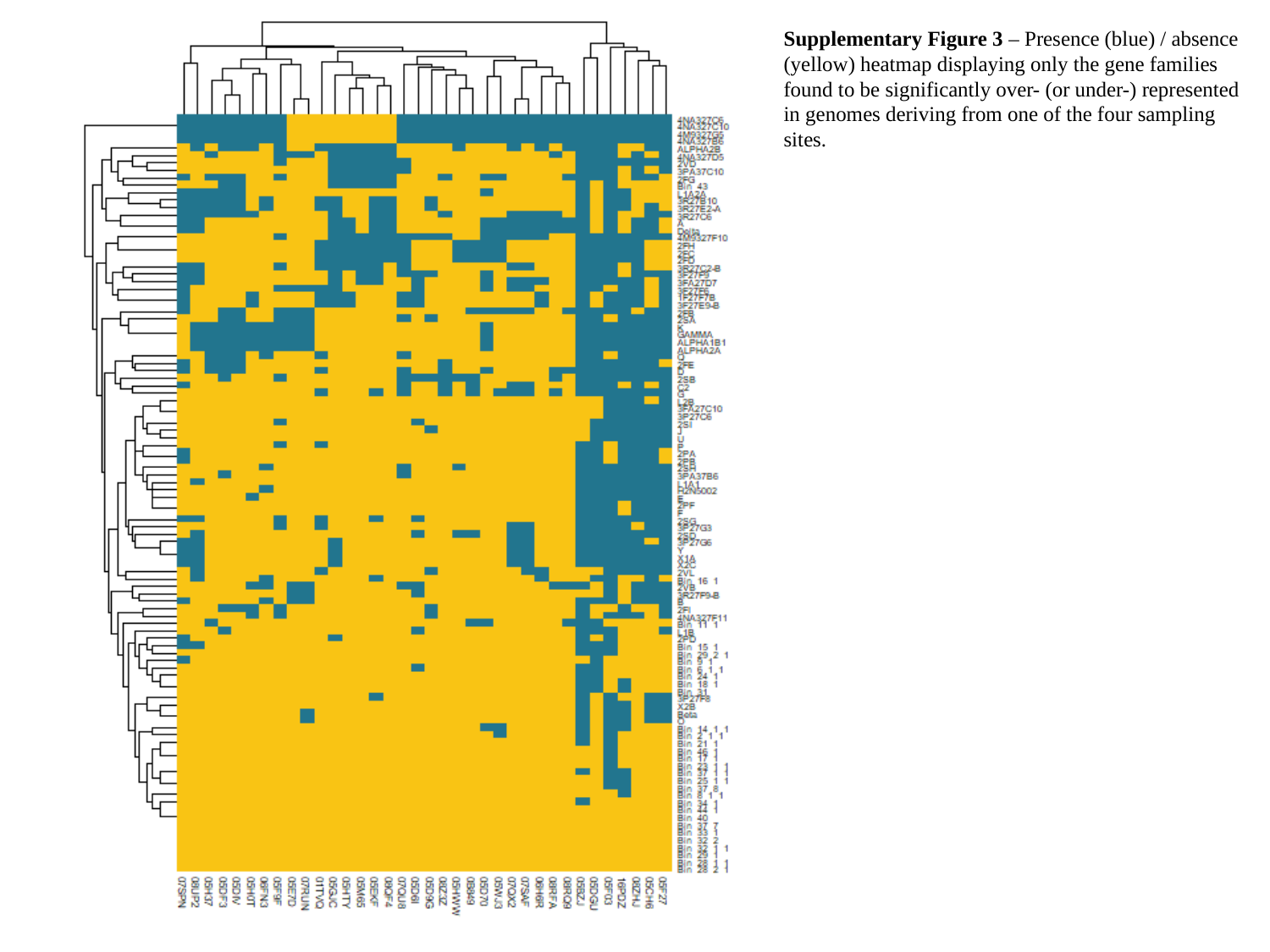

Supplementary Figure 3 – Presence (blue) / absence (yellow) heatmap displaying only the gene families found to be significantly over- (or under-) represented in genomes deriving from one of the four sampling sites.
