# Supplementary material for: Genomic and metagenomic insights into the microbial community of a thermal spring
Source: Microbiome. 2019 Jan 23;7:8. doi: 10.1186/s40168-019-0625-6 (PMC6343286; doi:10.1186/s40168-019-0625-6)
Supplement: Supplementary file 9 — Figure S4. Anvi’O plots displaying the clustering patterns of assembled contigs along with their coverage by each metagenome. Leftmost graph shows the contigs clustered only by sequence composition (i.e., tetrameric signature); rightmost graph clusters the contigs only by their differential coverage; middle graph, combination of differential coverage and tetrameric composition. (PPTX 829 kb) [file 40168_2019_625_MOESM9_ESM.pptx]

## Slide 1
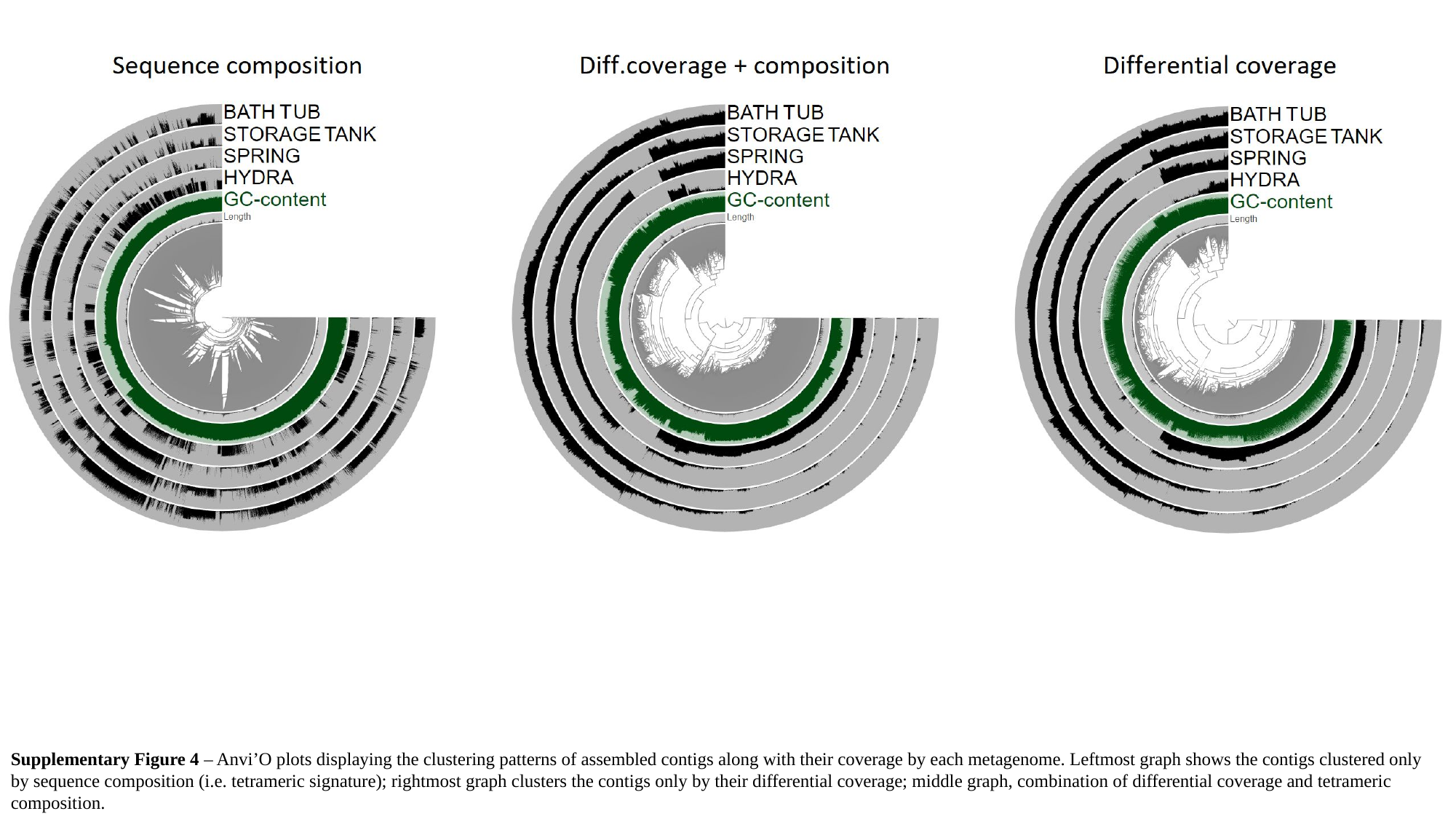

Supplementary Figure 4 – Anvi’O plots displaying the clustering patterns of assembled contigs along with their coverage by each metagenome. Leftmost graph shows the contigs clustered only by sequence composition (i.e. tetrameric signature); rightmost graph clusters the contigs only by their differential coverage; middle graph, combination of differential coverage and tetrameric composition.
